# A software‐based observational coding approach for evaluating paediatric dental pain, anxiety, and fear

**DOI:** 10.1111/ipd.13227

**Published:** 2024-07-12

**Authors:** Clare Bocklage, Raven Selden, Olivia Tumsuden, Eleanor Nanney, Caroline Sawicki, Allen Rapolla, Katelyn Cass, Jessica Lee, Jeannie Ginnis, Timothy Strauman, Christina Graves, Kimon Divaris, Eric Hodges, Laura Anne Jacox

**Affiliations:** ^1^ Orthodontics Group, Division of Craniofacial and Surgical Care, Adams School of Dentistry University of North Carolina Chapel Hill North Carolina USA; ^2^ Division of Biomedical Sciences, Adams School of Dentistry University of North Carolina Chapel Hill North Carolina USA; ^3^ DDS Program, Adams School of Dentistry University of North Carolina Chapel Hill North Carolina USA; ^4^ Division of Pediatrics and Public Health, Adams School of Dentistry University of North Carolina Chapel Hill North Carolina USA; ^5^ Department of Psychology and Neuroscience Duke University Trinity College of Arts and Sciences Durham North Carolina USA; ^6^ Department of Epidemiology, Gillings School of Global Public Health University of North Carolina Chapel Hill North Carolina USA; ^7^ School of Nursing University of North Carolina Chapel Hill North Carolina USA; ^8^ Present address: MyOrthodontist, Mount Airy Mount Airy North Carolina USA; ^9^ Present address: Summers Orthodontics Greenville South Carolina USA

**Keywords:** behavior, community paediatric dentistry, pain control, prevention, sedation

## Abstract

**Background:**

Dental practitioners desire non‐pharmacological methods to alleviate anxiety, fear, and pain in children receiving dental care; high‐quality evidence, however, is required to evaluate methods' efficacy.

**Aim:**

This study aimed to develop and validate an observation‐based coding approach (paediatric dental pain, anxiety, and fear coding approach [PAFCA]) to evaluate non‐pharmacological behavior management techniques for anxiety, fear, and pain.

**Design:**

Objective (video‐based) and subjective (self‐reported) anxiety, fear, and pain data were collected from a pilot clinical trial evaluating animal‐assisted therapy (AAT) in paediatric dentistry, in which 37 children aged 7–14 were assigned to AAT or control before dental treatment (restorations or extractions). A coding approach utilizing a codebook, a gold standard calibration video, and a user training guide was developed. Trained examiners coded the gold standard video for inter‐rater agreement, and masked, calibrated examiners analyzed videos using the Noldus Observer XT software.

**Results:**

A novel, software‐based coding approach was developed, with moderately high inter‐rater agreement. Using PAFCA, we found children reporting higher levels of pain, fear, and anxiety exhibited treatment‐interfering behaviors, including crying/moaning, attempts to dislodge instruments, and more upper and lower body movements.

**Conclusion:**

PAFCA shows promise as a reliable tool for assessing anxiety, pain, and fear in behavioral research for paediatric dentistry.

**Why this paper is important to paediatric dentists**

- PAFCA is a novel software‐based approach to behavioral coding of paediatric dental patients, which provides a comprehensive description of behaviors and experiences of children undergoing relatively invasive dental care. This method could ultimately lead to a better understanding of non‐pharmacological behavior management in paediatric dentistry.
- PAFCA integrates self‐report survey scales and simultaneous video measures over time, providing a detailed view of DA, DF, and pain during dental procedures.
- In the clinic, providers can identify behaviors shown here to be associated with DA, DF, and pain, and then adjust their clinical approach to alleviate discomfort and improve patients' experiences.

## 1 INTRODUCTION

Dental anxiety (DA) is the state of apprehension in anticipation of a negative dental experience.^1^ Over 50% of children report some level of DA.^2^ Patients affected by DA often exhibit uncooperative behavior and avoid preventative and restorative care.^1^ DA is associated with adverse health outcomes, including increased rates of decay, pain, extractions, infections, and emergency dental visits, and is correlated with a reduced quality of life.^2^, ^3^ For a majority of patients, DA develops in childhood due to traumatic experiences or vicarious learning from parents and can lead to lifelong DA.^2^, ^3^ Dental fear (DF), an in‐the‐moment response to perceived threatening stimuli, contributes significantly to DA development.^1^ Similarly, pain can further exacerbate negative experiences.^4^

To reduce DA and DF, effective behavioral management techniques must be employed during childhood to improve dental experiences.^5^ The American Academy of Pediatric Dentistry recommends pharmacological and non‐pharmacological behavior guidance techniques to deliver dental care to anxious children.^5^ Paediatric dentists choose techniques based on the patient's health history, dental needs, consequences of no treatment, emotional and intellectual development, and the parents' and dentist's preferences.^5^

Pharmacological behavior management (ie, sedation and general anesthesia) is often required for highly anxious or very young patients with advanced treatment needs—although paediatric sedation and general anesthesia are safe, they do carry risks that range from minor to very serious.^6^ Consequently, parents may prefer to avoid pharmacologic behavior management for dental treatment due to morbidity and mortality risks.^6^, ^7^ It is also important to acknowledge that costs for dental care with general anesthesia are significant, with average inflation‐adjusted expenditures of about $10 000 per case—an unfeasibly high cost for routine care.^8^ In the United States, 100 000–250 000 paediatric dental sedations occur annually.^7^ In North Carolina alone, 10 000 children under the age of 8 (15.8 per 1000) receive dental general anesthesia each year, with an annual expenditure of $16.7 M in 2015 ($21.1 M inflation‐adjusted), which has since risen, posing a major financial burden to private‐pay families and state Medicaid systems.^9^

Effective non‐pharmacological behavior guidance approaches are needed for anxious patients to ensure safe, cost‐effective care delivery with positive experiences. This demand has led clinicians and researchers to explore approaches such as animal‐assisted therapy (AAT), hypnosis, and distraction techniques using music, audio, audiovisual means, and toys.^10^, ^11^, ^12^, ^13^ To objectively evaluate the efficacy of behavior guidance techniques for DA and DF, there is a need for validated and reliable quantitative measures—and therein lies the opportunity to leverage technological advances in video observational coding and synchronous physiological monitoring.

Several behavioral evaluation scales have been developed for dentists to measure treatment‐interfering behavior and anxiety. A behavioral scale is a tool to quantitatively represent the level of a child's anxiety, fear, or pain. The most common is the observation‐based Frankl behavior rating scale due to its ease of clinical use and widespread adoption by paediatric dentists.^14^ A Frankl score, however, does not define specific behaviors for scoring and is subjective.^15^ Conversely, the Behavior Evaluation Scale (BES) and Behavior Profile Rating Scale (BPRS) are observation‐based assessments with definitive coded behaviors, used primarily in research due to their complexity and need for user calibration.^15^, ^16^ These coding schemes non‐continuously measure uncooperativeness during dental visits to gauge ease of care delivery, rather than patient anxiety, and were validated against the Frankl Scale.^14^ For example, the BPRS tracks 27 behaviors, weighted by how disruptive the behavior is to dental care; scoring is non‐continuous, using 3‐min segments to count behavior frequency.^14^ These behavioral rating scales were developed for direct observer scoring, and not for use with continuous, observational coding software or synchronous biometric data collection, ignoring valuable data on co‐occurrences between procedural events, patient behaviors, and physiologic changes. Continuous data provide a greater wealth of information, often lost in incremental scoring approaches.

**FIGURE 1:**
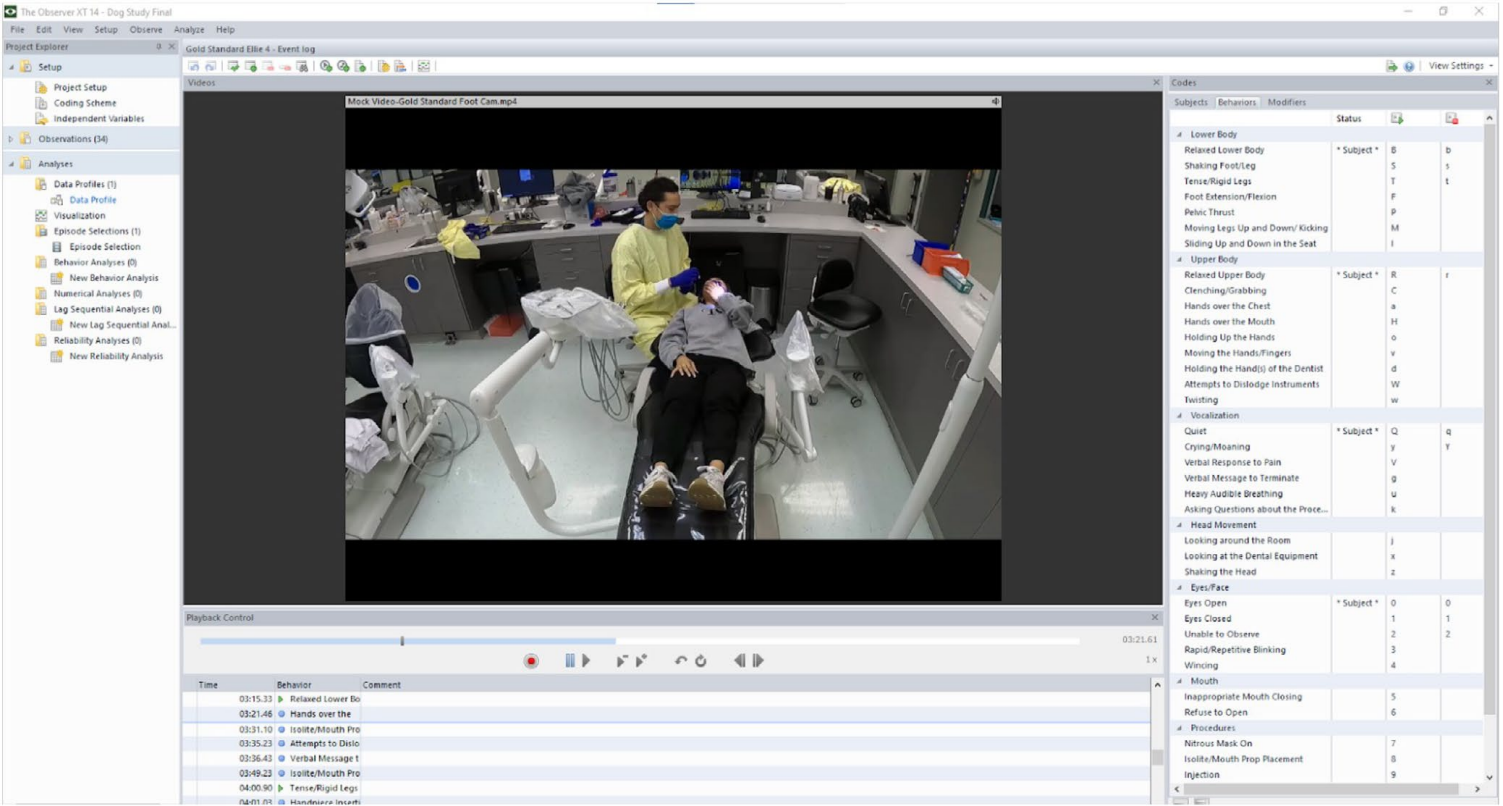
Noldus Observer XT with codebook. Video footage acquired by GoPro video camera and imported into Noldus Observer XT for behavior analysis.

To provide the research community with a metric for evaluating the efficacy of behavior management techniques on DA and DF, we developed a novel quantitative observational coding scheme using the video analysis software (Noldus Observer XT, Figure 1). We sought to validate it against self‐reported anxiety, fear, and pain scales. Our continuous coding approach, the paediatric dental pain, anxiety, and fear coding approach (PAFCA), was developed in concert with an ongoing pilot trial studying AAT. This prospective trial generated video and survey data from paediatric patients undergoing dental care, which were used to develop, test, and validate PAFCA. The overarching goal of this project is to offer an objective tool to quantify changes in DA, DF, and pain perception with continuous integration of data across video and biometric platforms.

## 2 MATERIALS AND METHODS

### 2.1 Data origination

Data utilized for developing this observational behavior coding method were derived from a prospective, controlled trial assessing whether AAT impacts physiologic measures and perceptions of anxiety, fear, and pain during paediatric dental visits (J. Massouda, N. Ghaltakhchyan, J. Judd, Unpublished Data). The study described here is not intended to evaluate the efficacy of AAT for reducing anxiety or pain, but rather utilized video and self‐report data from the AAT efficacy study to develop the PAFCA coding scheme. The study received human subjects' ethics approval (UNC IRB #19‐1911) and was registered with clinical.trials.gov (NCT04708028).

### 2.2 Overall study design

Potential participants were identified via chart review, selecting youths (7–14 years old) with a history of mild‐to‐moderate DA (36 Frankl 3–4, 1 Frankl 2) who were scheduled to receive dental treatment involving local anesthetic injections, without moderate‐to‐deep sedation in the Graduate Pediatrics Clinics at the University of North Carolina (UNC) Adams School of Dentistry (Table 1). This age group was selected to limit developmental differences between participants.^1^, ^3^ Informed consent from guardians and assent from participants were obtained.

**TABLE 1:** Inclusion/exclusion criteria.

Inclusion criteria	Exclusion criteria
Patients (all sexes, all genders) with stable physical health (ASA I–II) between the ages of 7 and 14 Frankl score of 2, 3, or 4 English and Spanish speakers Patients planned to receive dental treatment in the University of North Carolina Pediatric Dental Clinic, which includes an injection followed by a handpiece and/or forceps usage (eg, restoration/filling, stainless steel crown, or extractions)	Patients <7 years old and >15 years old Frankl score of 1 Non‐English and Non‐Spanish speakers Patients receiving oral sedation or general anesthesia Significant learning disability, developmental delay, or complex medical history, which complicates dental treatment, at the discretion of the study team Allergy to dogs and/or oral hygiene products (toothpaste and mouth rinse) Xerostomia (dry mouth) Previous traumatic experience with a dog and/or fear of dogs No patient assent and/or parental/guardian consent

A total of 37 paediatric DA patients (7–14 years old, *n* = 17 + AAT, *n* = 20 − AAT, Table 2) were consecutively enrolled and assigned to either a passive (quiet time) or active control group (coloring a dog picture) or the experimental intervention group (a standardized AAT session with a therapy dog). The AAT or control intervention occurred before the dental procedure.

**TABLE 2:** Sample demographics.

	Control (*n* = 20)	Animal therapy intervention (*n* = 17)
Age mean ± SD	9.65 ± 1.79	9.35 ± 2.09
Age range	7–13	7–14
Sex	40% Male (*n* = 8) 60% Female (*n* = 12)	35% Male (*n* = 6) 65% Female (*n* = 11)
Race	15% Black (*n* = 3) 5% Asian/Pacific Islander (*n* = 1) 20% Caucasian (*n* = 4) 60% Latin American (*n* = 12)	18% Black (*n* = 3) 0% Asian/Pacific Islander (*n* = 0) 6% Caucasian (*n* = 1) 76% Latin American (*n* = 13)
Ethnicity	60% Hispanic (*n* = 12) 40% Non‐Hispanic (*n* = 8)	76% Hispanic (*n* = 13) 24% Non‐Hispanic (*n* = 4)
Received an injection	85% (*n* = 17)	88% (*n* = 15)
Received nitrous oxide	70% (*n* = 14)	88% (*n* = 15)
Dental procedure performed during study visit	55% Restorative (*n* = 11) 9 resin restorations 2 sealants 4 SSC 40% Extraction (*n* = 8) 5% No procedure, only injection (*n* = 1), aborted due to uncooperative behavior	65% Restorative (*n* = 11) 7 resin restorations 3 sealants 3 SSC 35% Extraction (*n* = 6)
Average procedure (video) duration ± SD	35.55 ± 15.69 min	37.41 ± 19.05 min
Range of procedure (video) duration	13–75 min	12–77 min
Average Frankl score^a^ ± SD	3.65 ± 0.59	3.76 ± 0.44
Frankl score	70% Frankl 4 (*n* = 14) 25% Frankl 3 (*n* = 5) 5% Frankl 2 (*n* = 1)	76% Frankl 4 (*n* = 13) 24% Frankl 3 (*n* = 4) 0% Frankl 2 (*n* = 0)
Parent‐reported anxiety disorder	15% Yes (*n* = 3) 85% No (*n* = 17)	0% Yes (*n* = 0) 100% No (*n* = 17)
Fear of dental visit	20% Very afraid (*n* = 4) 25% Somewhat afraid (*n* = 5) 30% Only a little afraid (*n* = 6) 25% Not afraid at all (*n* = 5)	0% Very afraid (*n* = 0) 6% Somewhat afraid (*n* = 1) 35% Only a little afraid (*n* = 6) 59% Not afraid at all (*n* = 10)

^a^ Frankl score was determined by the paediatric dentist who treated the patient before the study visit and was used for screening purposes.

### 2.3 Measures

**FIGURE 2:**
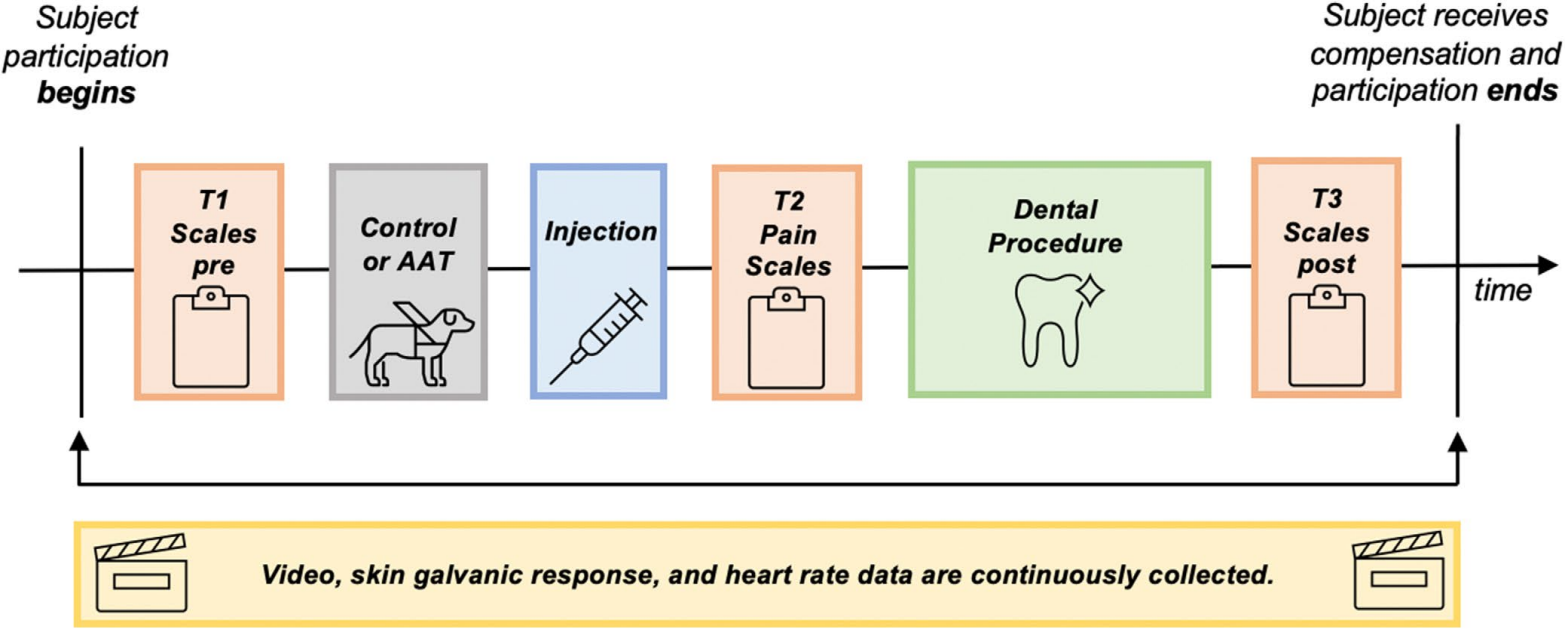
Study visit summary: Subject participation begins with consent and a synchronous video (by GoPro), heart rate, and skin galvanic response recording (by Shimmer 3 GSR+ or Polar Verity Sense Devices). Participants fill out a pre‐procedural survey of fear, anxiety, and pain scales (Survey Time 1: Wong–Baker FACES Pain Rating Scale, Modified Child Dental Anxiety Scale [MCDAS], and Children's Fear Survey Schedule—Dental Subscale [CFSS‐DS]). A standardized therapy dog interaction occurs for experimental participants; active controls color a dog picture. Local anesthetic is injected, followed by the pain scale (Survey Time 2: Wong–Baker FACES Pain Rating Scale). The dental procedure occurs and includes restorative (resin restorations, sealants, or stainless steel crowns) or surgical (extractions) care. After the procedure, participants will fill out a post‐procedure survey about the experience including fear, anxiety, and pain scales (Survey Time 3: Wong‐Baker FACES Pain Rating Scale, MCDAS, and CFSS‐DS). Study participation concludes.

Synchronous video data were recorded throughout the visit (using wireless GoPro Hero9 Cameras) (GoPro Inc., San Mateo, CA, USA) to evaluate body movements. Cameras recorded .mp4 files in 10‐min segments, which were compiled using the Camtasia software (TechSmith, Okemos, MI, USA) to create a continuous .mp4 file for import into Noldus Observer XT for coding and analysis (Noldus Information Technology, Wageningen, the Netherlands). The Observer XT software allowed for quantitative, behavioral coding to record events for analysis of observational data, and it enabled the synchronization of events from different data streams for statistical analysis.^17^ Pre‐ and post‐procedural survey data on perceived anxiety, fear, pain and expectations were obtained. These survey scales included the Modified Child Dental Anxiety Scale (MCDAS), Wong–Baker FACES Pain Rating Scale, and Children's Fear Survey Schedule—Dental Subscale (CFSS‐DS), and were validated for ages 8–12, 3–18, and 5–14 years, respectively.^18^, ^19^, ^20^, ^21^ These scales were administered before (Time 1) and after (Time 3) the procedure, with the Wong–Baker FACES Pain Rating Scale administered a third time immediately after the anesthetic injection (Time 2) (Figure 2). Figure 2 summarizes the study visit.

### 2.4 Codebook development and refinement

Validated scales and coding schemes for paediatric DA were used to develop an initial codebook.^1^, ^15^, ^22^, ^23^, ^24^ A codebook contains the descriptions of movements and actions that can characterize a person's behavior. A hybrid list of codes was created from the BES and BPRS, combining relevant codes in Noldus Observer XT.^1^, ^15^, ^16^, ^22^, ^23^, ^24^ Additional codes were added by experienced paediatric dentists, to fill gaps in potentially observable behaviors. Similar to the BES, the PAFCA list of codes was divided by body region, including upper body, lower body, vocalizations, head, eyes, face, and mouth. Codes were also categorized as state or point events (Noldus Software terminology). Sustained behaviors are state events, such as “crying” or “eyes closed”, and are measured by duration in seconds. Brief, momentary actions, such as verbal responses to pain or wincing, are point events and counted by event occurrence. Procedure codes were added to allow coordination of events in the video (ie, injection, instrument insertion) with changes in behavioral codes. Point, state, and procedural codes are listed in Table 3 with code definitions, as used in Noldus.

**TABLE 3:** Codebook and definitions—Study codebook organized by code category, code name, event type, and code definition.

Code category	Code	Event	Code definition
Lower body	Relaxed lower body (− Anxiety^a^)	Initial state	Still/relaxed lower body
	Shaking foot/leg^b^	State	Movement of foot/leg side to side Repetitive, distinct/prominent shaking from left to right, not a residual movement
	Tense/rigid legs	State	Contraction of leg muscles keeping legs rigid or straight
	Foot extension/flexion	Point	Extension or flexion of either one or both feet
	Pelvic thrust	Point	Pushing off heels or feet to elevate pelvic region
	Kicking^c^ /moving legs up and down^b^	Point	Either one or both feet kicking/raising up, down, or away from body
	Sliding up and down in seat	Point	Using lower body to push up or move down in the chair
Upper body	Relaxed upper body (− Fear^a^)	Initial state	Still/relaxed upper body/extremities Clasped hands are considered relaxed unless the knuckles are white/clenched If no other codes for the upper body are occurring, this code should be on
	Attempts to dislodge instruments^c^ (+ Pain^a^)	Point	Grabbing instruments to remove from face/mouth
	Clenching/grabbing (+ Pain^a^)	Point	Clenching fists or grabbing chair tightly or white knuckles
	Hand(s) over the mouth^b^	Point	Placing hand(s) over mouth or covering mouth
	Hands over the chest^b^	Point	Hands held in position over chest Release of hands over chest is not a separate code
	Holding up the hand(s)^b^	Point	Raising hand(s) Can be in opposition to treatment, to attempt to stop or to signal pain This movement differs from moving the hands/fingers because it involves movement of the arms
	Moving the hand(s)^b^ (+ Pain^a^)	Point	Movement of fingers, wrists If there is arm movement, refer to “holding up the hands”
	Holding the hand(s) of dentist^b^	Point	Grabbing the hand(s) of the dentist Includes pulling the dentists' hand(s) away, or stopping treatment from being administered
	Twisting	Point	Twisting of the torso or shoulders
	Inappropriate sitting up in chair	Point	Attempts to sit up from supine position during appointment at an inappropriate time
Vocalization	Quiet (− Anxiety, − Fear, − Pain^a^)	Initial state	No verbal communication
	Moaning/crying^c^ (+ Anxiety, + Fear^a^)	State	Constant soft whimper Unintelligible moans Weeping/sobbing with altered breathing or speech
	Verbal response to pain^b^ (+ Fear^a^)	Point	Verbal expression of pain in the form of speech Eg, ouch, ow, and it hurts
	Verbal message to terminate^c^	Point	Verbal communication to stop procedure or verbal disapproval of procedure Eg, No, I don't like it, Stop
	Heavy audible breathing	Point	Increase in depth, sound, or rate of breathing without prompting
	Asking questions about the procedure^b^	Point	Asking questions in relation to what is going to happen
Head movement	Shaking the head^b^	Point	Moving the head side to side Can communicate disapproval
Eyes/face	Eyes open	Initial state	Eyes open
	Eyes closed^c^	State	Eyes closed
	Unable to observe	State	Eyes are not observed due to wearing protective eyewear or blocking from dentist or assistant
	Looking around the room^b^ (− Fear^a^)	Point	Surveying the room looking at people present or instruments without being requested to do so by the dentist
	Looking at the dental equipment^b^ (− Fear^a^)	Point	Looking at the assistant tray/suction or dentist's tray or handpieces without being requested to do so by the dentist This code includes when the dentist is holding the equipment
	Rapid repetitive blinking^b^	State	Rapid blinking due to surprise, pain, or fear
	Wincing^b^	Point	Either partial closure or full closure of the eyes due to contraction of periorbital muscles of facial expression Include stiffening of the face
Mouth	Inappropriate mouth closing^c^	Point	Purposely closing mouth during dental procedure
	Refuse to open^c^	Point	Refusal to open mouth for procedure
Procedures	Nitrous mask on	Point	Placement of nasal hood for intranasal nitrous oxide delivery
	Injection	Point	Local anesthetic administration
	Isolite/mouth prop placement	Point	Insertion of instruments used to keep patient's mouth open during procedure
	Extraction instrument insertion	Point	Insertion of elevators or forceps for extraction of teeth
	Handpiece insertion	Point	Insertion of dental drill to complete restorative dentistry

^a^ Behavior positively or negatively correlated with anxiety, fear, or pain (specified in parentheses), as evaluated by MCDAS, CFSS‐DS, or Wong–Baker FACES Pain Rating Scale, respectively.

^b^ Taken or modified from Behavior Evaluation scale.

^c^ Taken or modified from the Behavior Profile Rating scale.

Once a preliminary codebook was complete, five videos were imported into Noldus for testing and revision. Repeated consensus‐coding meetings were held between senior investigators, including an observational research expert (E.H.), to revise the code list and definitions for clarity. A “gold standard” calibration video was created of a mock patient visit to train study team members; a user training guide was also created (Appendix S1, Video S1). Four examiners underwent training. Examiners were provided with a detailed codebook, with in‐depth descriptions of each code. During training, coders were also shown a comprehensive training video, with examples of what would and would not be considered a code under the PAFCA. These trained examiners coded the gold standard video; inter‐rater reliability was assessed within Noldus using confusion matrix correlation tables. User feedback was collected on how to improve the training guide and coding scheme. The training guide and codebook were further revised, with clarified definitions and exclusion of frequently misused codes. When two codes were used incorrectly and interchangeably with low reliability, the two codes were collapsed into a single code that encompassed both behaviors (eg, shaking foot and shaking leg). The codebook was finalized by senior authors at a final consensus‐coding meeting. The mock patient video was then reanalyzed by the trained examiners, using the revised codebook and updated training guide, yielding high inter‐ and intra‐rater reliabilities. All patient videos were coded, with every fifth video (20%) coded by all coders to ensure high inter‐rater reliability. Coding of each video began with first watching it from start‐to‐finish and was then followed by re‐watching the video with detailed coding. Coders limited their coding sessions to 2 h to avoid fatigue.

### 2.5 Statistical analysis

The ‘gold standard’ calibration video was used for an inter‐ and intra‐rater reliability assessment via Cohen's kappa statistics.^25^ Data obtained from behavior codes and self‐report scales were analyzed descriptively and for outliers. Based on outlier analyses, two observations were excluded from further analysis, leaving a final sample of 37 subjects (*n* = 20 − AAT, *n* = 17 + AAT). We also created behavioral class variables based on body region (ie, upper or lower body), and these, along with the individual behaviors, were included in correlational analyses with the MCDAS, Wong–Baker FACES Pain Rating Scale, and CFSS‐DS to assess criterion validity. Due to the small sample size (*N* = 37) and non‐normal distribution of most variables, associations were evaluated using nonparametric (Spearman's *ρ*) correlation tests and group comparisons (Wilcoxon test). Both nominal (*p* < .05) and suggestive (*p* < .1) statistical associations were considered. Descriptive, correlational, and comparative analyses were performed using JMP Pro (Version 17.2.0; JMP Statistical Discovery LLC, Cary, NC, USA). Inter‐rater reliability was assessed using kappa statistics in the Noldus Observer XT Software, and average kappa values indicated moderate agreement, ranging from the fair to almost perfect (Range: .25–.83; Mean: .51).^25^

## 3 RESULTS

### 3.1 Sample description

Most participants (*n* = 34) received local anesthetic injections before procedures requiring dental handpieces or forceps. For five patients planned for restorative treatment, the provider decided not to inject local anesthetic (*n* = 3 − AAT, *n* = 2 + AAT). The intervention and control groups were well‐matched by age, sex, and Frankl score (Table 2). The AAT exposure group had slightly more Black and Hispanic participants (AAT: 18% Black and 76% Hispanic; Control: 15% Black and 60% Hispanic). Nearly all participants had documented mild‐to‐moderate DA based on Frankl scores, and consequently, the majority (ie, 78%) received nitrous oxide.

### 3.2 Reliability testing

The “gold standard” calibration video was used for inter‐ and intra‐rater reliability assessment via Cohen's kappa statistic (Video S1).^25^ After training, new coders reached an average inter‐rater reliability kappa of .80. Cohen's kappa values of ≥.60 or greater indicate agreement, and .80 is considered very strong agreement.^25^ The final codebook had an average inter‐rater reliability of .51, suggesting a moderately reliable and repeatable scheme.^25^ The change in inter‐rater reliability kappa from .80 during training to .51 with experimental videos could be due to the increased complexity of behaviors during a real visit as compared to the mock gold standard video used for training.

### 3.3 Codebook description and validation

**FIGURE 3:**
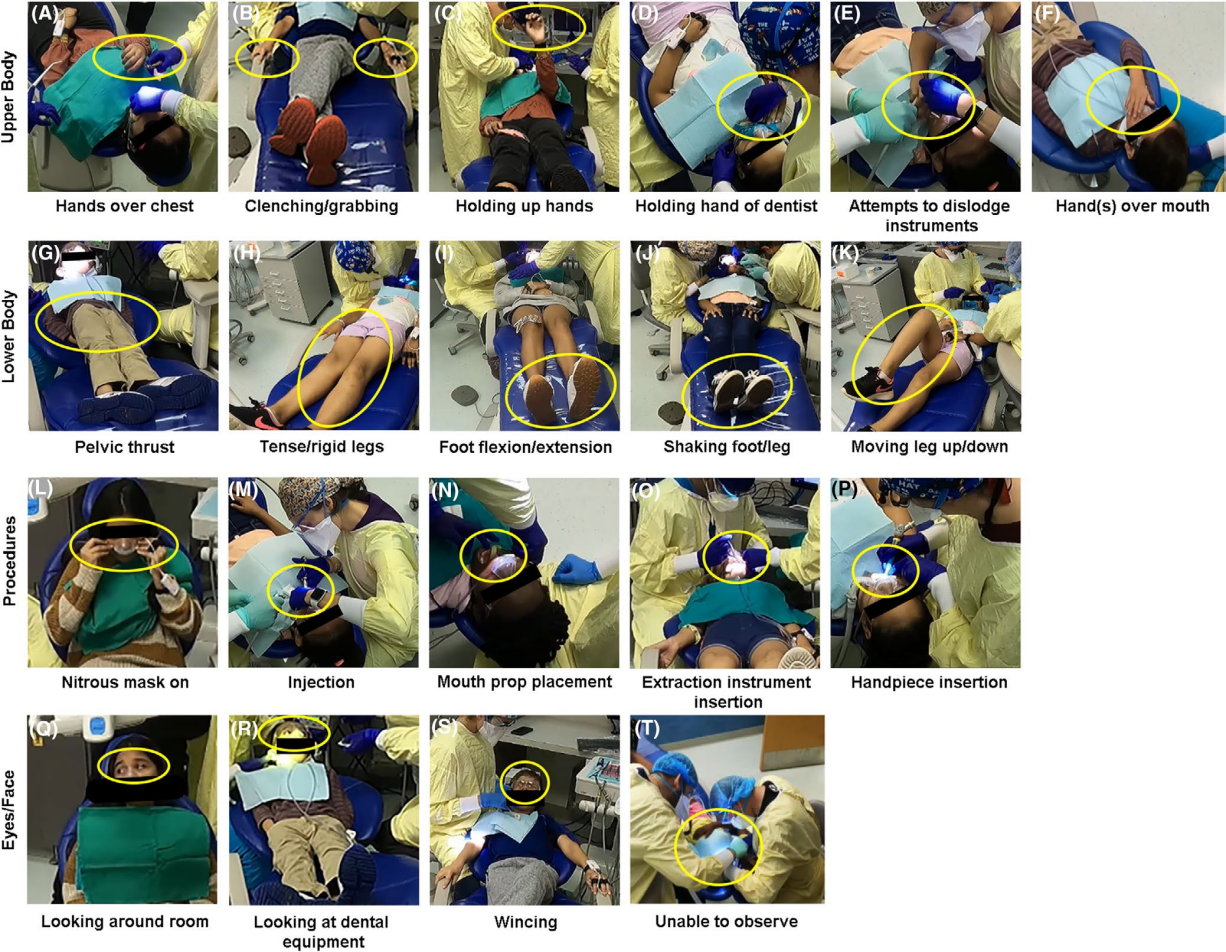
Representative images of codes organized by category. Categories include the lower body, upper body, procedural steps, and eyes/face. The code is highlighted with a yellow oval. Code definitions are detailed in Table 3. Video examples of codes are included in Video S1. A user guide is included in Appendix S1.

The final codebook of state, point, and procedure codes is presented in Table 3, with still photograph and video examples of codes shown in Figure 3. A user training guide is included in Appendix S1. Relationships between self‐report measures, behavior classes, procedures, and coded behaviors are outlined in Tables 4, 5, 6, 7, 8, 9, 10. Associations between coded behaviors and children's self‐reported pain, anxiety, and fear scales were used to validate PAFCA codes relative to patients' self‐perceptions. Because the sample was small (*N* = 37), both significant (*p* < .05) and borderline significant (*p* < .1) correlations were noted.

**TABLE 4:** Correlations among behavior classes and coded behaviors.

Behavior 1	Behavior 2	Correlation coefficient (Spearman's *ρ*)	*p* Value
Total lower body	Holding up hand(s)/moving hand(s)	.4747	.003**
	Clenching/moving hand(s)	.4272	.0084**
	Hands over chest/holding up the hand(s)/hand(s) over mouth	.3214	.0524*
	Shaking foot/tense rigid legs/foot extension flexion	.8934	<.001**
	Twisting	.3102	.0617*
	Moving legs up and down/kicking	.6308	<.001**
	Total upper body	.5622	<.001**
	Relaxed upper body	.3623	.0276**
	Relaxed lower body	−.5042	.0015**
Total upper body	Holding up hand(s)/moving hand(s)	.9525	<.001**
	Hands over chest/holding up the hand(s)/hand(s) over mouth	.6575	<.001**
	Clenching/moving hand(s)	.8319	<.001**
	Holding the hand(s) of the dentist	.4660	.0037**
	Sliding up and down in seat	.3882	.0176**
	Moving legs up and down/ kicking	.2995	.0717*
	Shaking foot/tense rigid legs/foot extension flexion	.4542	.0047**
Total vocalizations	Hands over chest/holding up the hand(s)/hand(s) over mouth	.495	.0018**
	Quiet	−.7396	<.001**
	Crying/moaning	.7556	<.001**
	Verbal response to pain	.5904	<.001**
	Verbal message to terminate	.3669	.0255**
	Relaxed lower body	−.2966	.0746*
Total eyes	Looking around the room, looking at the dental equipment	.9848	<.001**
	Sliding up and down in seat	.3492	.0341**
Total face	Sliding up and down in seat	.3886	.0174**
	Attempts to dislodge instruments	.4787	.0027**
	Verbal response to pain	.3118	.0603*

*Note*: Correlations with *p* > .1 are not shown.

*p < .1 borderline significance. **p < .05 significance convention.

**TABLE 5:** Correlations among coded behaviors.

Behavior 1	Behavior 2	Correlation coefficient (Spearman's *ρ*)	*p* Value
Holding up hand(s)/moving hand(s)	Clenching/moving hand(s)	.7855	<.001**
	Sliding up and down in seat	.4053	.0128**
	Holding the hand(s) of the dentist	.4654	.0037**
	Shaking foot/tense rigid legs/foot extension flexion	.3581	.0296**
Clenching/moving hand(s)	Pelvic thrust	.285	.0873*
	Shaking foot/tense rigid legs/foot extension flexion	.303	.0683*
	Holding the hand(s) of the dentist	.3231	.0511*
Hands over chest/holding up the hand(s)/hand(s) over mouth	Verbal response to pain	.4528	.0049**
	Holding the hands of the dentist	.4302	.0079**
	Sliding up and down in seat	.2893	.0823*
	Shaking foot/tense rigid legs/foot extension flexion	.3242	.0502*
	Relaxed lower body	−.4358	.007**
Looking around the room, looking at the dental equipment	Sliding up and down in seat	.3703	.0241**
	Moving legs up and down/ kicking	.2817	.0912*
	Pelvic thrust	.3828	.0194**
Twisting	Pelvic thrust	.4472	.0055**
Sliding up and down in seat	Inappropriate mouth closing	.3886	.0174**
	Verbal response to pain	.3191	.0542*
Crying/moaning	Verbal message to terminate	.3524	.0324**
	Verbal response to pain	.2777	.0961*
	Quiet	−.8323	<.001**
Shaking foot/tense rigid legs/foot extension flexion	Relaxed upper body	.3655	.0261**
	Relaxed lower body	−.60	<.001*
	Moving legs up and down/ kicking	.3585	.0294**
Quiet	Relaxed lower body	.4860	.0023**

*Note*: Correlations with *p* > .1 are not shown.

*p < .1 borderline significance. **p < .05 significance convention.

**TABLE 6:** Correlations among self‐report measures.

Scale 1	Scale 2	Correlation coefficient (Spearman's *ρ*)	*p* Value
CFSS‐DS 1 Sum	MCDAS 1 Sum	.6672	<.001**
	CFSS‐DS 2 Sum	.7383	<.001**
	MCDAS 2 Sum	.6367	<.001**
	Wong‐Baker FACES 1	.3629	.0273**
	Wong‐Baker FACES 3	.3516	.0328**
MCDAS 1 Sum	MCDAS 2 Sum	.5916	<.001**
	CFSS‐DS 2 Sum	.6101	<.001**
	Wong–Baker FACES 1	.4207	.0095**
	Wong–Baker FACES 3	.4118	.0113**
Wong–Baker FACES 1	Wong–Baker FACES 3	.4043	.0131**
CFSS‐DS 2 Sum	MCDAS 2 Sum	.8302	<.001**
	Wong–Baker FACES 2	.3099	.0619*
	Wong–Baker FACES 3	.3340	.0433**
Wong–Baker FACES 2	Wong–Baker FACES 3	.3068	.0647**

*Note*: Correlations with *p* > .1 are not shown.

Abbreviations: CFSS‐DS, Children's Fear Survey Schedule—Dental Subscale Questionnaire; MCDAS, Modified Child Dental Anxiety Scale Questionnaire.

*p < .1 borderline significance. **p < .05 significance convention.

**TABLE 7:** Correlations among behavior classes and self‐report measures.

Behavior class	Scale	Correlation coefficient (Spearman's *ρ*)	*p* Value
Total vocalizations	CFSS‐DS 2 Sum	.4208	.0095**
	MCDAS 2 Sum	.3731	.0229**
Total eyes	Wong–Baker FACES 1	−.2954	.0759*
	Wong–Baker FACES 3	−.4940	.0019**
Total upper body	Wong–Baker FACES 2	.3329	.0441**

*Note*: Correlations with *p* > .1 are not shown.

*p < .1 borderline significance. **p < .05 significance convention.

**TABLE 8:** Correlations among self‐report measures and coded behaviors.

Scale	Behavior	Correlation coefficient (Spearman's *ρ*)	*p* Value
CFSS‐DS 1 Sum	Relaxed upper body	−.3363	.0419**
MCDAS 1 Sum	Relaxed lower body	−.3216	.0522*
	Quiet	−.2797	.0936*
Wong–Baker FACES 1	Looking around the room, looking at the dental equipment	−.2789	.0946*
	Attempts to dislodge instruments	.3277	.0477*
Wong–Baker FACES 2	Quiet	−.2983	.073*
	Clenching/moving hand(s)	.3259	.049**
CFSS‐DS 2 Sum	Quiet	−.3623	.0276**
	Crying/moaning	.2839	.0886**
	Verbal response to pain	.3572	.03**
MCDAS 2 Sum	Quiet	−.5152	.0011**
	Crying/moaning	.4196	.0097**
	Looking around the room, looking at the dental equipment	−.4648	.0038**

*Note*: Correlations with *p* > .1 are not shown.

*p < .1 borderline significance. **p < .05 significance convention.

**TABLE 9:** Correlations among procedures and self‐report measures.

Procedure	Scale	Correlation coefficient (Spearman's *ρ*)	*p* Value
Extraction instrument insertion	Wong–Baker FACES 3	.5221	<.001**
Handpiece insertion	Wong–Baker FACES 2	−.3790	.0207**
	Wong–Baker FACES 3	−.4593	.0042**

*Note*: Correlations with *p* > .1 are not shown.

*p < .1 borderline significance. **p < .05 significance convention.

**TABLE 10:** Correlations among procedures and coded behaviors.

Procedure	Behavior	Correlation coefficient (Spearman's *ρ*)	*p* Value
Nitrous mask on	Relaxed upper body	.4318	.0076**
	Total face	.3122	.06*
	Inappropriate mouth closing	.3122	.06*
Injection	Relaxed upper body	.2947	.0766*
	Total vocalizations	.4264	.0085**
	Crying/moaning	.3621	.0277**
Handpiece insertion	Relaxed upper body	.4241	.0089**
	Quiet	.42	.0096**
	Crying/moaning	−.3156	.0571*
	Total eyes	.2829	.0898*
	Looking around the room, looking at the dental equipment	.2753	.0991*
Isolite/mouth prop placement	Sliding up and down in seat	−.5161	.0011**
	Total vocalizations	.2841	.0884*
	Total eyes	−.3521	.0326
	Looking around the room, looking at the dental equipment	−.3676	.0252**
Extraction instrument insertion	Total eyes	−.4637	.0038**
	Looking around the room, looking at the dental equipment	−.4613	.0041**
	Twisting	−.2981	.0731*

*Note*: Greater handpiece insertions positively correlated with looking around the room and at the dental equipment, having a relaxed upper body, and being quiet. Conversely, increased handpiece insertions were less likely to result in crying/moaning. Isolite/mouth prop placement was negatively correlated with eye codes, looking codes, and sliding up and down in the seat, but positively correlated with vocalizations. Meanwhile, extraction instrument insertions were negatively correlated with twisting, eye codes, and looking around the room and at the dental equipment. Correlations with *p* > .1 are not shown.

*p < .1 borderline significance. **p < .05 significance convention.

Self‐reported scales were evaluated for correlation with one another, at each time point (Visit Time points: 1, 2, and 3, Figure 2, Table 6). Self‐report measures for fear, anxiety, and pain correlated with one another; specifically, CFSS‐DS (ie, fear) correlated strongly with MCDAS (ie, anxiety). Children who were more anxious and fearful at the start of the procedure tended to be so throughout. Patients who reported higher levels of pain pre‐procedure tended to report higher pain post‐procedure. More anxious and fearful subjects also tended to experience more pain.

**FIGURE 4:**
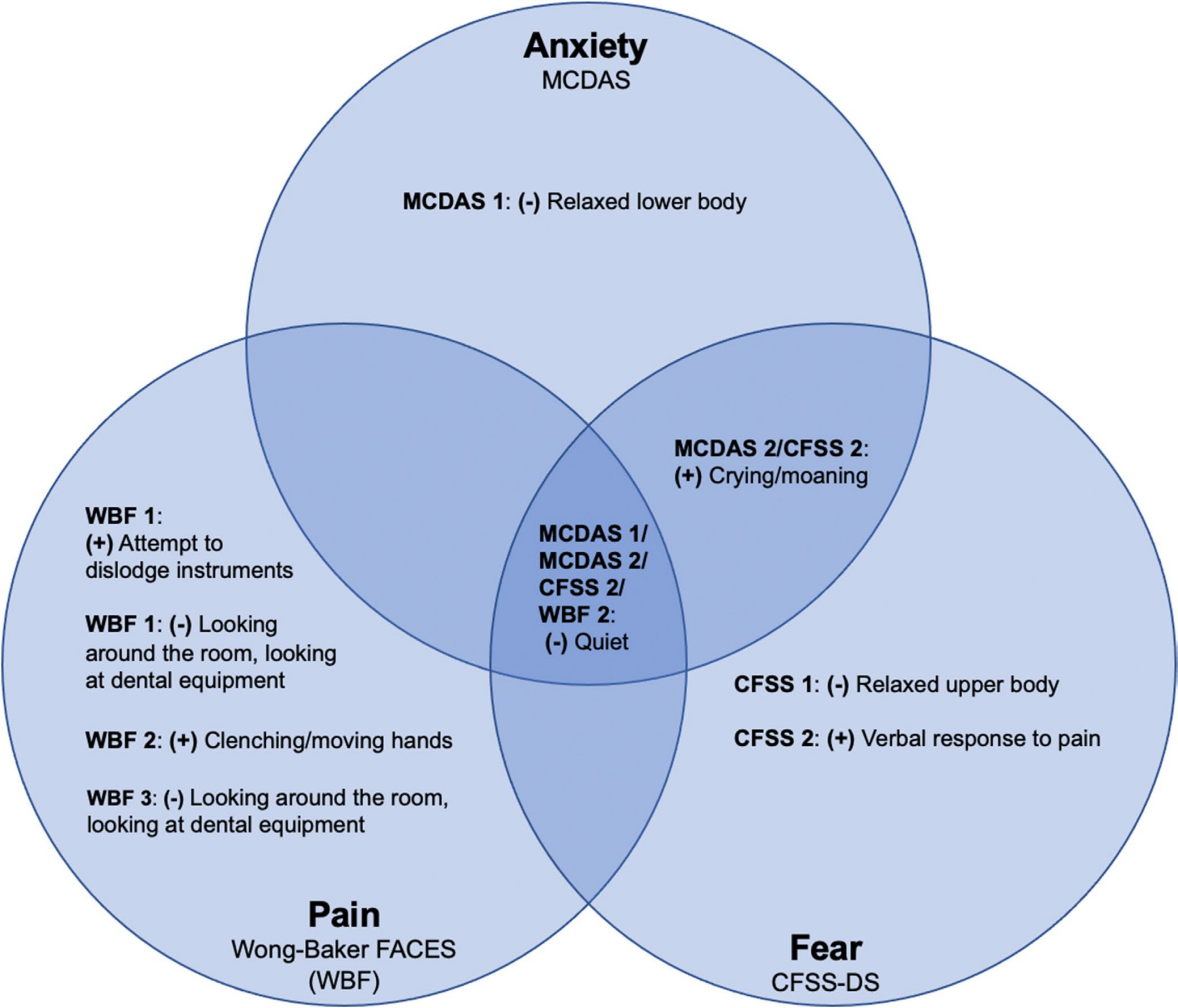
Correlation Venn diagram of self‐report measures and coded behaviors (Tables 7, 8, 9, 10). This demonstrates behavior correlations with anxiety, fear, or pain, as reported by Modified Child Dental Anxiety Scale (MCDAS), Children's Fear Survey Schedule—Dental Subscale (CFSS‐DS), and Wong‐Baker FACES Pain Rating Scale, respectively. Self‐report measures were collected at three time points: pre‐procedural (Wong‐Baker FACES 1, MCDAS 1, and CFSS‐DS 1), post‐injection (Wong‐Baker FACES 2), and post‐procedural (Wong‐Baker FACES 3, MCDAS 2, and CFSS‐DS 2).

Relationships between self‐report measures, coded behaviors, behavior classes, and procedures were also evaluated (Figure 4, Tables 7, 8, 9, 10).

#### 3.3.1 Correlations between MCDAS and behaviors (Figure 4, Tables 7 and 8)

A higher incidence of vocalizations was associated with increased post‐procedural anxiety. More specifically, the incidence of moaning and crying increased with higher anxiety levels. Patients who reported increased pre‐procedural anxiety tended to display less occurrences of relaxed lower body and being quiet. Additionally, patients who were more anxious after the procedure tended to be less quiet and were more likely to cry/moan during the procedure.

#### 3.3.2 Correlations between CFSS‐DS and behaviors (Figure 4, Tables 7 and 8)

Children who experienced increased pre‐procedural fear were less likely to have a relaxed upper body. Crying, moaning, and verbal responses to pain increased, and the average amount of quiet time decreased with greater post‐procedural fear.

#### 3.3.3 Correlations between Wong–Baker FACES and behaviors (Figure 4, Tables 7 and 8)

Patients who reported higher levels of pain at baseline attempted to dislodge instruments more than patients who reported lower pain levels. The occurrence of clenching, grabbing, and moving the hands correlated with higher levels of pain mid‐procedure. Finally, looking at the dental equipment and looking around the room negatively correlated with pre‐ and post‐procedural pain levels. Patients who experienced greater extraction instrument insertions were more fearful post‐procedure, whereas patients who experienced greater handpiece insertions were less likely to report pain during and after the procedure.

#### 3.3.4 Correlations between coded behaviors (Tables 4 and 5)

Some upper and lower body movements were positively correlated with one another, such as clenching and moving the hands with shaking foot/leg, tense and rigid legs, and foot extension/flexion. Particularly, uncooperative behaviors, such as twisting and pelvic thrusting, were highly correlated. Conversely, children with still lower bodies tended to be quieter.

#### 3.3.5 Correlations between procedures, behaviors, and self‐report measures (Tables 9 and 10)

Children receiving nitrous tended to have a relaxed upper body but were more likely to display inappropriate mouth closing. Children who received more injections showed not only increased incidence of vocalizations and crying/moaning and increased post‐procedure pain scores but also had a more relaxed upper body.

## 4 DISCUSSION

PAFCA was used to continuously code the behavior of children receiving dental care. With moderately high inter‐rater reliability, PAFCA is reproducible across coders after a brief training exercise, demonstrating its feasibility and usefulness for research. This new method is novel as it utilizes continuous coding while having the capacity to incorporate self‐reported and physiological measures. This allows researchers to develop an unobtrusive, comprehensive picture of paediatric dental anxiety, fear, and pain during patient care to evaluate the efficacy of behavioral interventions.

It is important to note that although anxiety and fear are distinct from one another, they are related. Anxiety and fear exist on a continuum and are physiological and psychological responses that help individuals to protect themselves from perceived threats. Anxiety is an anticipatory emotional state that allows someone to prepare for future situations, whereas fear is an in‐the‐moment arousal response to a perceived imminent danger. Both fear and anxiety can significantly impact when and how patients seek dental care.^2^ Practically, in the clinic, it can be challenging to delineate between DA, DF, and pain based on observational and qualitative data. Imaging studies have revealed significant overlap in brain regions responsible for modulating anxiety and fear, suggesting differentiation based on threat immediacy and response duration may not be straightforward.^26^

Here, we included DA (MCDAS) and DF (CFSS‐DS) scales to reflect both emotions. Similarly, we evaluated self‐reported pain levels using the Wong‐Baker FACES Pain Rating Scale, as DF, DA, and pain can collectively contribute to the “negative emotional load” of dental patients.^1^ Our scales had high but non‐equivalent correlations, indicating that the scales are related but measure distinct constructs delineating DF, DA, and pain via self‐responses (J. Massouda, N. Ghaltakhchyan, J. Judd, Unpublished Data). Coded behaviors also correlated with certain scales and not others, indicating particular behaviors may be more associated with anxiety, fear, or pain (Figure 4).

In this study, we evaluated state DA rather than trait‐based anxiety, using PAFCA. State anxiety is characterized by its transient nature, arising acutely in response to specific adverse stimuli, whereas trait anxiety is considered inherent to one's personality.^27^ Akin to state anxiety, DF shares similarities as a response triggered by immediate, threatening stimuli.^26^, ^27^ As this study did not incorporate a validated self‐report measure aimed specifically at state DF and DA (such as Visual Analog or Facial Image scales), and DF and DA were not measured mid‐procedure due to clinical time constraints, we are limited in our ability to distinguish state‐versus‐trait DA and DF.^28^, ^29^

Correlations between coded behaviors and self‐reported scales paint a descriptive picture of children at the dentist. Anxious or fearful children tended to have more vocalizations such as crying and moaning. Additionally, children who received more injections were more likely to vocalize, cry, or moan. After children received an injection, they were more likely to report higher pain levels and exhibit behaviors such as clenching/grabbing. Crying and moaning were also correlated with higher self‐report fear and anxiety scales. Overall, higher rates of upper and lower body movements were positively correlated, whereas relaxed lower bodies were correlated with children being quiet.

Many of the PAFCA behavioral codes were derived from the BPRS and BES instruments, which were validated against the Frankl Scale as indicators of patient compliance.^15^, ^24^ Validating relative to Frankl has limitations, as this scale is not associated with specific behaviors and may not correlate with anxiety and pain in children who are well‐behaved regardless of stress and pain level.^15^ Frankl is also a subjective score of behavioral compliance, instead of self‐reported anxiety or pain, which is likely more relevant to the development of DA and avoidance behavior.^21^ BPRS, originally described by Melamed et al.,^16^ includes 27 behaviors in which patients were coded in 3‐min, non‐continuous increments. The BPRS‐ and BES‐coded behaviors are weighted depending on how disruptive the behavior is to dental care, indicating that these scales are for measuring the feasibility of care delivery, rather than quantifying a child's fear or anxiety.^14^ Associating the PAFCA codes with self‐report scales is a step toward validating behaviors as representative of a patient's perceived anxiety, fear, and pain. This shifts the focus from scoring clinical compliance, a measure most relevant to a provider, to tracking patient experience, a metric that may be more indicative of current and future DA and avoidance.

PAFCA aims to balance usability with comprehensiveness for researchers. Videos from paediatric dental visits can be subtly collected with wall‐mounted cameras, to avoid interrupting patient care and inconveniencing providers, while offering high‐quality, continuous data for later analysis. The coding data can then be combined with self‐report scales and physiological data to allow for statistical analysis within Noldus, to evaluate correlations within and across groups (J. Massouda, N. Ghaltakhchyan, J. Judd, Unpublished Data). PAFCA can be employed to evaluate the effectiveness of interventions aimed at reducing DA, DF, and perceived pain in paediatric patients, such as behavioral therapy, AAT, pharmacological interventions, or distraction techniques. Taken together, PAFCA is a novel tool for assessing dental pain, anxiety, and fear in paediatric patients and could be used for research purposes to identify efficacious behavior modification strategies to reduce negative emotions and treatment‐interfering behaviors, and ultimately improve care. In live clinical settings, providers can identify behaviors shown here to be associated with DA, DF, and pain, and then adjust their clinical approach to alleviate discomfort and improve patients' experiences.

Although PAFCA utilizes codes included in the BES and BPRS, it is a continuous coding approach to collect comprehensive, quantitative datasets from videos for research purposes. Prior schemes such as BES and BPRS were non‐continuous and did not utilize software tools for data collection. In addition to tracking code frequencies or duration, the Noldus software enables statistical analyses for tabulations of rater reliability, code confusion, and correlation matrices, lending greater insights to video‐based data.^17^ Although the PAFCA code list is longer than the BES and BPRS instruments, the behaviors were user‐tested for reliability and stratified into categories for ease of coding. Synchronous physiologic data (ie, heart rate and galvanic sweat response) can also be aligned with video data, to associate events with changes in biometric measures, adding further insight into patients' experiences. Furthermore, the high level of inter‐rater reliability indicates that the codebook is user‐friendly and understandable; with more extended training and experience, raters may be able to achieve higher inter‐rater reliability.^25^

PAFCA is a novel software‐based approach to behavioral coding of paediatric dental patients, validated against self‐reported pain, fear, and anxiety. It describes the behaviors and experiences of children undergoing dental care. PAFCA is unique in its ability to integrate survey scales, simultaneous video, and physiological measures over time, providing a comprehensive view of anxiety, fear, and pain during invasive dental procedures. PAFCA was designed to be used continuously with video of a paediatric dental visit, so it remains minimally invasive to patient care while allowing for assessment of dental anxiety for research seeking to evaluate behavior modification approaches.

### 4.1 Limitations

A limitation is the small sample size resulting in sparse data on uncommon behaviors. Despite having DA, most participants were Frankl 4, such that participants exhibited few severe behavior codes (eg, pelvic thrusting and twisting). Severe, noncompliant codes remain in the codebook, as we anticipate them being needed for children with lower Frankl scores, though these codes could not be validated in this initial investigation and must be pursued in future studies. Additionally, nitrous oxide use likely reduced the overall frequency of codes and the occurrence of more severe codes. Excluding participants receiving nitrous use, however, would skew results away from a naturalistic dataset that is representative of true clinical environments. With a smaller sample, we are more likely to find spurious correlations with outliers skewing data. With a larger sample, codes that demonstrate borderline significance will likely have adequate power. The ongoing study evaluating AAT will provide a larger sample for additional validation of codes through self‐report scales and will allow us to compare control with experimental (AAT) interventions. A future direction is validating PAFCA behavioral codes against synchronous physiologic measures such as heart rate or sweat response.

Dental treatments vary in invasiveness. Procedures involving injections, extractions, and handpieces evoke significant negative reactions linked to DF, DA, and pain; as a result, we focused on these treatments.^1^ Dental care, however, includes other less invasive preventative and diagnostic procedures such as radiographs and oral examinations. Further validation encompassing a wider range of treatment interventions is necessary and currently ongoing.

One additional limitation was the drop in average inter‐rater reliability from substantial to moderate agreement (.80–.51) between the gold standard and the experimental videos. The gold standard video was used for training to ensure proper use of the codebook, potentially making it clearer to code than real behaviors exhibited during dental visits. Children may display multiple behaviors simultaneously during actual visits, increasing coding complexity. Despite this, inter‐rater reliability remained moderately high.

## AUTHOR CONTRIBUTIONS

L.A.J., K.C., E.H., and K.D. conceived of the ideas; C.B., A.R., J.G., and J.L. collected the data; L.A.J., E.H. and K.D. supervised and guided the methodology; C.B., R.S., O.T., and E.N. performed video coding; C.B., R.S., A.R., L.A.J., and E.H. assembled and revised the code book; C.B., R.S., E.H., and L.A.J. performed data analysis and interpretation; L.A.J., C.G., T.S., and K.D. also contributed to results interpretation and visualization; C.B. and R.S. led the writing of the initial draft; all authors contributed to manuscript review and editing; and L.A.J. funded and supervised the project.

## FUNDING INFORMATION

This work was supported by the Southern Association of Orthodontics Research Award (to A.R. and K.C.). This work was also supported by the American Association of Orthodontists Foundation (AAOF) Resident Research Award (to A.R. and K.C.). The project described was supported by the National Center for Advancing Translational Sciences (NCATS), National Institutes of Health (NIH), through Grant Award Number UL1TR002489, through the National Institute of Dental and Craniofacial Research (NIDCR) with a K08 grant (K08DE030235) (to L.A.J.) and with an R03 grant (R03DE032768) (to L.A.J.). The content is solely the responsibility of the authors and does not necessarily represent the official views of the NIH.

## CONFLICT OF INTEREST STATEMENT

The authors have no conflicts of interest to declare.

## INSTITUTIONAL REVIEW BOARD

This research was approved by the Institutional Review Board of The University of North Carolina in Chapel Hill (IRB #19‐1911). The data used in observational coding were collected as part of a registered clinical trial (NCT04708028, registered January 2021).

## Supporting information


Appendix S1

VideoS1

## Data Availability

The data supporting the findings of this study are available within the article and its supplementary materials.
